# Inhibition of HDAC6 by Tubastatin A reduces chondrocyte oxidative stress in chondrocytes and ameliorates mouse osteoarthritis by activating autophagy

**DOI:** 10.18632/aging.202736

**Published:** 2021-03-19

**Authors:** Zhonghai Shen, Kang Ji, Zhenhai Cai, Chenglong Huang, Xiaojun He, Hongwei Xu, Gang Chen

**Affiliations:** 1 Department of Orthopedic Surgery, The Second Affiliated Hospital of Jiaxing University, Jiaxing, China

**Keywords:** histone deacetylase, Tubastatin A, osteoarthritis, autophagy

## Abstract

The aim of this study was to determine the effect of HDAC6 inhibition using the selective inhibitor Tubastatin A (TubA) on the regulation of tert-butyl hydroperoxide (TBHP)-treated chondrocytes and a mouse OA model. Using conventional molecular biology methods, our results showed that the level of HDAC6 increases both in the cartilage of osteoarthritis (OA) mice and TBHP-treated chondrocytes *in vitro*. TubA treatment effectively inhibits the expression of HDAC6, attenuates oxidative stress, reduces the level of apoptotic proteins to maintain chondrocyte survival, and suppresses the extracellular matrix (ECM) degradation. In addition, our results also revealed that HDAC6 inhibition by TubA activates autophagy in chondrocytes, whereas the protective effects of TubA were abolished by autophagy inhibitor intervention. Subsequently, the positive effects of HDAC6 inhibition by TubA were also found in a mouse OA model. Therefore, our study provide evidence that HDAC6 inhibition prevents OA development, and HDAC6 could be applied as a potential therapeutic target for OA management.

## INTRODUCTION

Osteoarthritis (OA) is one of the most prevalent age-related joint degeneration diseases among elderly individuals and is characterized by cartilage damage, synovial membrane inflammation and subchondral bone sclerosis, which results in persistent joint pain and functional disability in the affected patients, bringing great challenges to the quality of life and healthcare costs [1]. As the only resident cells, chondrocytes play a vital role in maintaining cartilage homeostasis and function and are regarded as a potent target for the treatment of OA [2, 3]. The pathological progression of OA in chondrocytes involves multiple pathological factors, including activation of inflammatory response, accumulation of reactive oxygen species, cell apoptosis and degradation of the extracellular matrix [4, 5]. Though the current treatment for OA is limited to inflammatory and pain intervention in clinics, pharmacological management or molecular method targeting to inhibit pathological response might be feasible for maintaining chondrocyte homeostasis and deferring the progression of joint degeneration [6–8].

The homeostasis of acetylation and deacetylation of lysine residues is a critical mechanism for maintaining normal physiological function. Histone deacetylase (HDAC) is one of the deacetylase proteins and regulates various biological functions by eliminating acetyl from proteins [9]. As a member of type II-b HDAC family, HDAC6 is the only enzyme that is characterized by two functional catalytic domains, primarily expressed in the cytoplasm and specifically catalyses the deacetylation of non-histone substrates of proteins [10]. The aberrant expression and functional structure of HDAC6 can cause a series of chromosome configuration reconstructions and changes, subsequently affecting the transcription regulation and leading to the development of diseases [11–13]. Intriguingly, HDAC6 has emerged as a potent target for the treatment of several diseases and has proven to be associated with sufficient efficacy. Numerous studies have revealed that HDAC6 is required for oncogenic cell transformation and tumour cell movement and invasion, and these studies have indicated that HDAC6 inhibition could improve the efficacy of anticancer treatment [14, 15]. In addition, HDAC6 inhibition also exhibits therapeutic effects on chronic kidney disease [16], heart injury [12], and spinal cord injury [17]. However, the role of HDAC inhibition in osteoarthritis has not been elucidated well.

Particularly, autophagy is a catabolic-related and lysosome-dependent process for maintaining cell homeostasis through recycling the dysfunction cellular organelles and redundant misfolded proteins and reactive oxygen species [18]. The basal level of autophagy is essential for cartilage tissue morphogenesis and chondrocyte homeostasis, while autophagy is dysfunction in the senile chondrocytes of OA [19, 20]. Existing evidence suggested that restoring the autophagy function is beneficial for the clearance of accumulation of reactive oxygen species and inflammatory mediators, which has been regarded as a potential strategy for maintaining chondrocyte survival and preventing the progression of OA [21, 22]. Intriguingly, numerous studies demonstrated that HDAC6 is a major regulator of autophagy and HDAC6-mediated autophagy is involved in the tumour cell destination under the administration of radiation and chemotherapy [23–25]. Based on previous studies, we reasoned that HDAC6 inhibition may be an advantageous mechanism for chondrocyte homeostasis. However, the role of HDAC6 in the autophagy of chondrocytes and the potential effect on OA remains rarely mentioned.

As HDAC6 exhibited the biological functions discussed above, a variety of small molecule inhibitors have been discovered for the treatment of the disease [26]. Tubastatin A (TubA), a potent and high selective HDAC6 inhibitor, has been reported to provide a therapeutic effect for several diseases [17, 27, 28]. Here, we evaluated whether HDAC6 is involved in OA development, and whether HDAC6 inhibition by TubA plays an available role in maintaining chondrocyte survival and preventing extracellular matrix (ECM) degradation. Collectively, by performing conventional molecular methods, our results revealed that treatment with TubA significantly ameliorates osteoarthritis and inhibits the level of HDAC6 in chondrocytes, which results in activation of autophagy, cell survival and reduction of ECM degradation. Thus, our results provide considerable evidence that HDAC6 inhibition by TubA may be a feasible strategy for the management of OA.

## RESULTS

### HDAC6 is increased in OA mice

**Figure 1:**
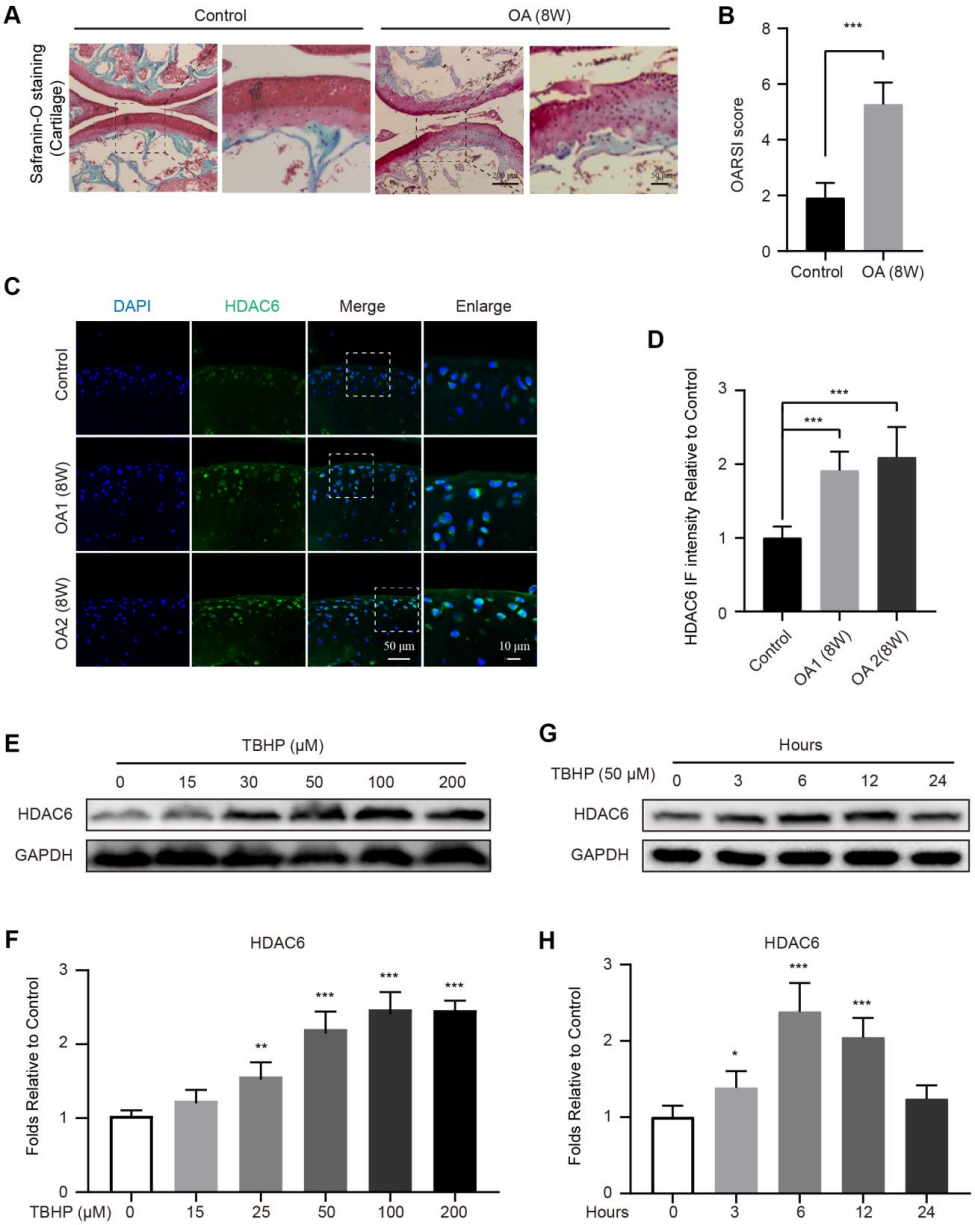
**Upregulation of HDAC6 in mouse knees and chondrocytes.** (**A**, **B**) Typical Safranin O staining of the cartilage and subchondral cortical bone in each group of mice, scale bar = 200 μm, scale bar (enlarged) = 50 μm. (**C**, **D**) IF staining and quantification of the changes in HDAC6 in control and OA mice, scale bar = 50 μm, scale bar (enlarged) = 10 μm. (**E**, **F**) Western blotting and quantification of HDAC6 in chondrocytes subjected to different concentrations of TBHP for 12 hours. (**G**, **H**) Western blotting and quantification of HDAC6 in chondrocytes subjected to 50 μM of TBHP in a time-dependent manner. N = 5, GAPDH was the loading control, significance: *P<0.05, **P<0.01, ***P<0.001.

To establish an OA model, mice were performed by surgical destabilization of the medial meniscus (DMM). After 8 weeks, the cartilages were stained with Safranin O, and the results showed that DMM group exhibits the devastative superficial articular cartilage (Figure 1A, 1B). Subsequently, the expression of HDAC6 in cartilages were evaluated in the Control and OA groups, the results of immunofluorescence (IF) staining revealed that the intensity of HDAC6 was significantly increased in 8 weeks after the mouse OA modelling was performed (Figure 1C, 1D). In addition, to further evaluate the change of HDAC6 in cells, we separately cultured primary chondrocytes and found that the level of HDAC6 is gradually upregulated under tert-butyl hydroperoxide (TBHP) stimulation in a dose-dependent manner (Figure 1E, 1F). Meanwhile, HDAC6 was also increased when chondrocytes were subjected to TBHP treatment in a time-dependent manner and reached the peak at 6 hours after TBHP treatment (Figure 1G, 1H). Therefore, these data indicated that HDAC6 is increased in chondrocytes and may be involved in the progression of OA.

### HDAC6 inhibition reduces chondrocyte apoptosis

**Figure 2:**
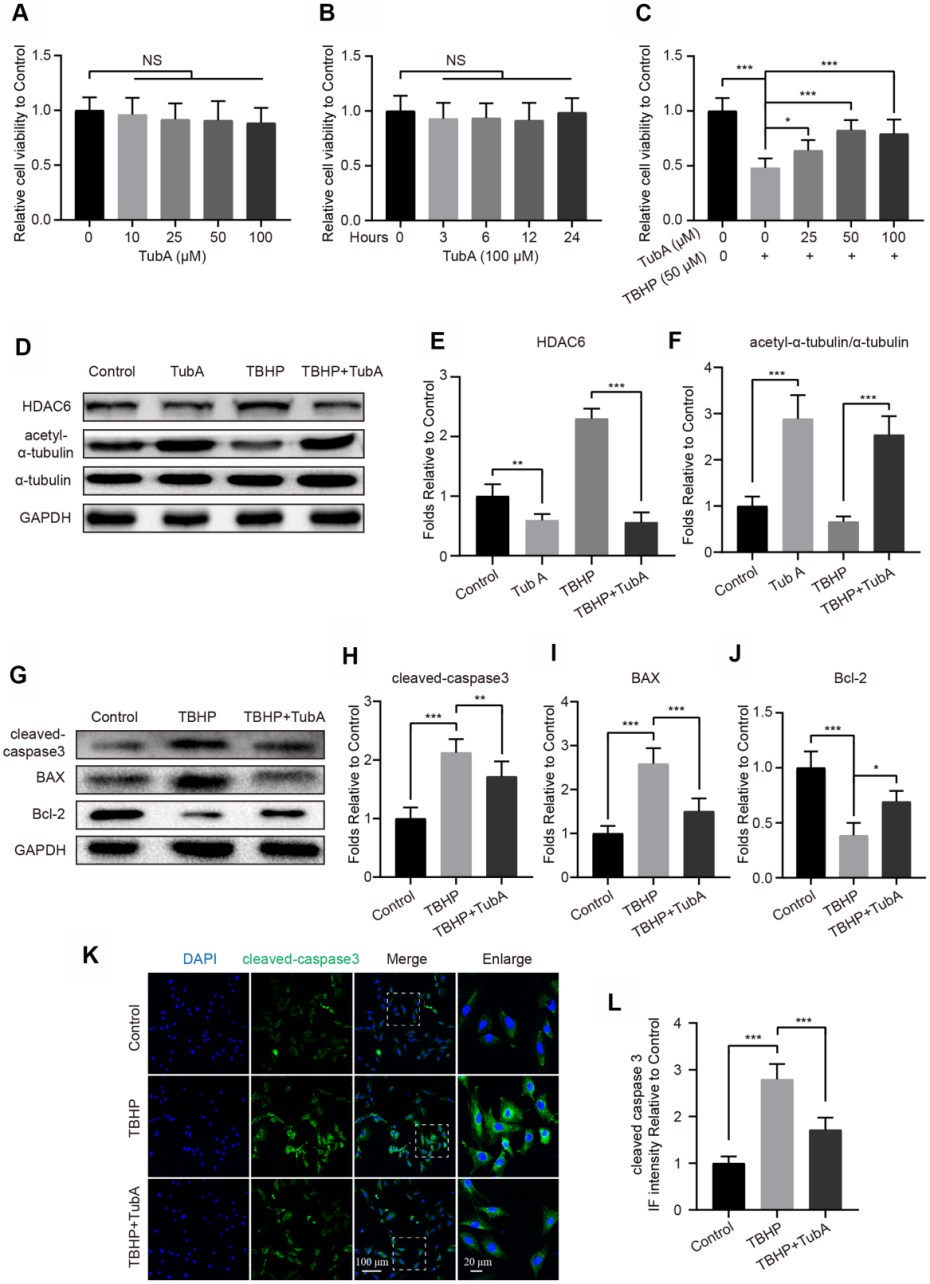
**HDAC6 selective inhibitor TubA prevents TBHP-induced apoptosis in chondrocytes.** (**A**) The cell viability of chondrocytes at 24 hours after different concentrations of TubA treatment. (**B**) The cell viability of chondrocytes treated with TubA at a concentration of 100 μM in a time-dependent manner. (**C**) The cell viability of chondrocytes at 24 hours after co-treatment of TBHP and TubA for 6 hours. (**D**–**F**) Western blotting and quantification of HDAC6 and acetyl-α-tubulin in each group. Chondrocytes were treated TBHP or/and TubA for 6 hours. (**G**–**J**) Western blotting and quantification of apoptotic markers in each chondrocyte group as above. (**K**, **L**) IF staining and quantification of cleaved caspase 3 in each chondrocyte group as above, scale bar = 100 μm, scale bar (enlarged) = 20 μm. N = 5, GAPDH was the loading control, significance: *P<0.05, **P<0.01, ***P<0.001.

To determine the effect of HDAC6 on chondrocytes, TubA was selected for specificity in inhibiting HDAC6 expression. First, we evaluated the toxicity of TubA on chondrocytes by the cell counting kit-8 (CCK8) assay. The results showed that TubA treatment presents no obvious cytotoxicity when the concentration is 100 μM at 12 hours (Figure 2A). The cell viability analysis in a time-dependent manner also showed no significant cytotoxicity from beginning to the end of TubA treatment (Figure 2B). In addition, TubA at the concentration 50 μM significantly reverses the decreased cell viability of chondrocytes under the TBHP treatment (Figure 2C). Therefore, TubA at the concentration 50 μM was used as the treatment concentration. This concentration of TubA effectively inhibits the expression of HDAC6 and enhances the level of acetyl-α-tubulin in chondrocytes with or without TBHP treatment (Figure 2D–2F). Meanwhile, the effect of TubA on apoptosis in mouse chondrocytes subjected to TBHP was evaluated by Western blotting and IF. As shown in Figure 2G–2J, the decreased expression of cleaved caspase3 and Bax and the enhanced expression of Bcl2 were found in chondrocytes with TubA treatment after TBHP stimulation. Similarly, the IF staining of cleaved caspase3 illustrated that TBHP-treated chondrocytes show a decreased intensity of cleaved caspase3 after TubA treatment (Figure 2K, 2L). In addition, TUNEL staining also showed that TubA treatment decreases the proportion of cellular apoptosis after TBHP stimulation (Supplementary Figure 1A, 1B). Thus, the above results suggest that TubA exerts an anti-apoptotic effect on mouse chondrocytes.

### HDAC6 inhibition attenuates oxidative stress in chondrocytes

**Figure 3:**
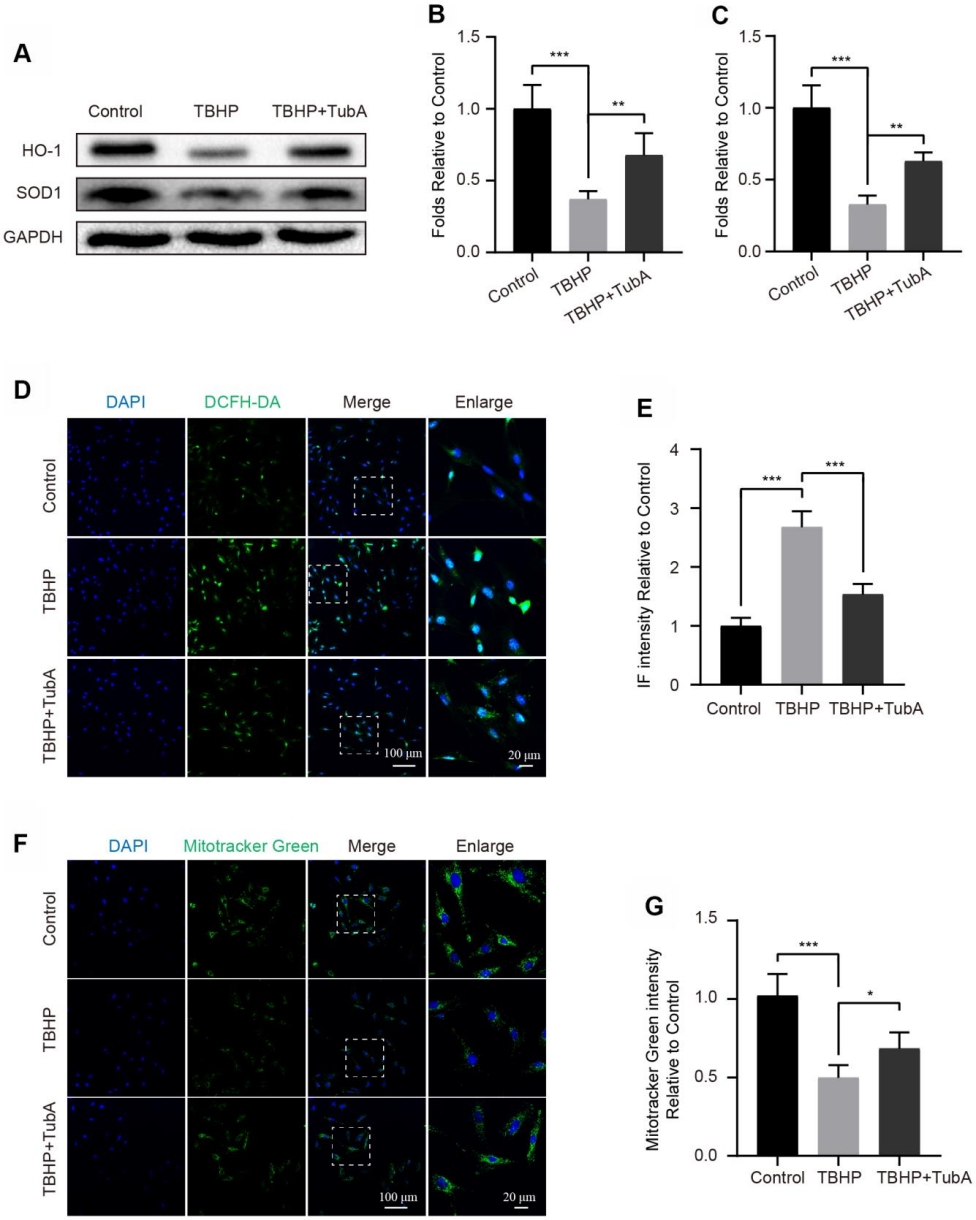
**TubA attenuates TBHP-induced oxidative stress in chondrocytes.** (**A**–**C**) Western blotting and quantification of HO-1 and SOD1 in each group. Chondrocytes were treated TBHP or/and TubA for 6 hours. (**D**, **E**) Staining with DCFH-DA and quantification in each chondrocyte group as above, scale bar = 100 μm, scale bar (enlarged) = 20 μm. (**F**, **G**) Staining with Mitotracker green and quantification in each chondrocyte group as above, scale bar = 100 μm, scale bar (enlarged) = 20 μm. N = 5, GAPDH was the loading control, significance: *P<0.05, **P<0.01, ***P<0.001.

The oxidative stress is one of the critical factors in the development of OA [29]. Here, we evaluated whether TubA is sufficient to attenuate cellular oxidative stress after TBHP stimulation. The Western blotting analysis indicated that TubA treatment effectually upregulated the level of SOD1 and HO-1. Both of them are important enzymes related to counteraction of oxidative stress (Figure 3A–3C). Consistence with Western blotting, the results of the ROS Assay Kit further confirmed that TubA remarkably decreased the intensity of DCFH-DA (Figure 3D, 3E). In addition, TubA treatment also enhanced the fluorescence intensity of Mitotracker Green in TBHP-treated chondrocytes (Figure 3F, 3G). Collectively, these data indicate that TubA is beneficial for reducing oxidative stress in chondrocytes.

### HDAC6 inhibition ameliorates TBHP-induced ECM degradation in chondrocytes

**Figure 4:**
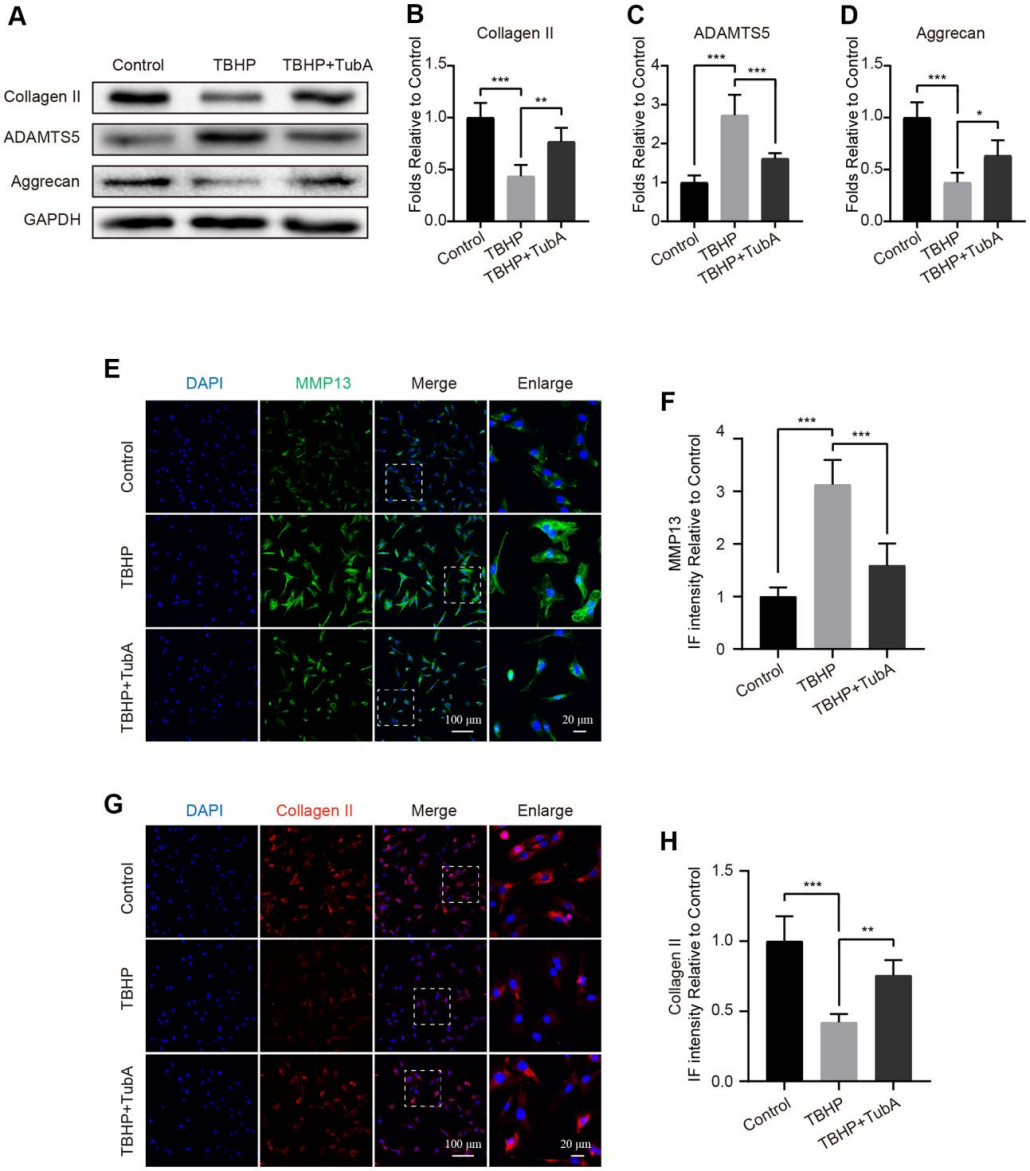
**Inhibition of HDAC6 by TubA represses ECM degradation in chondrocytes.** (**A**–**D**) Western blotting and quantification of Collagen II, ADAMTS5 and Aggrecan in each group. Chondrocytes were treated with TBHP or/and TubA for 6 hours. (**E**, **F**) IF staining and quantification of MMP13 in each chondrocyte group as above, scale bar = 100 μm, scale bar (enlarged) = 20 μm. (**G**, **H**) IF staining and quantification of Collagen II in each chondrocyte group as above, scale bar = 100 μm, scale bar (enlarged) = 20 μm. N = 5, GAPDH was the loading control, significance: *P<0.05, **P<0.01, ***P<0.001.

To determine whether HDAC6 inhibition by TubA is a contribution to the ECM synthesis, we detected the levels of several markers of ECM by Western blotting and IF. The results from Western blotting indicated that administration of TBHP significantly reduced the level of Collagen II and Aggrecan but increased the expression of ADAMTS5. In contrast, TubA reversed the above undesirable effects resulting from TBHP (Figure 4A–4D). Likewise, results from IF. Respectively, stained with Collagen II (Figure 4E, 4F) and MMP13 (Figure 4G, 4H) are in accordance with the Western blot analysis. Overall, these results demonstrated that TubA acts in a beneficial role in regulating ECM synthesis and metabolism.

### TubA enhances the level of autophagy in chondrocytes

**Figure 5:**
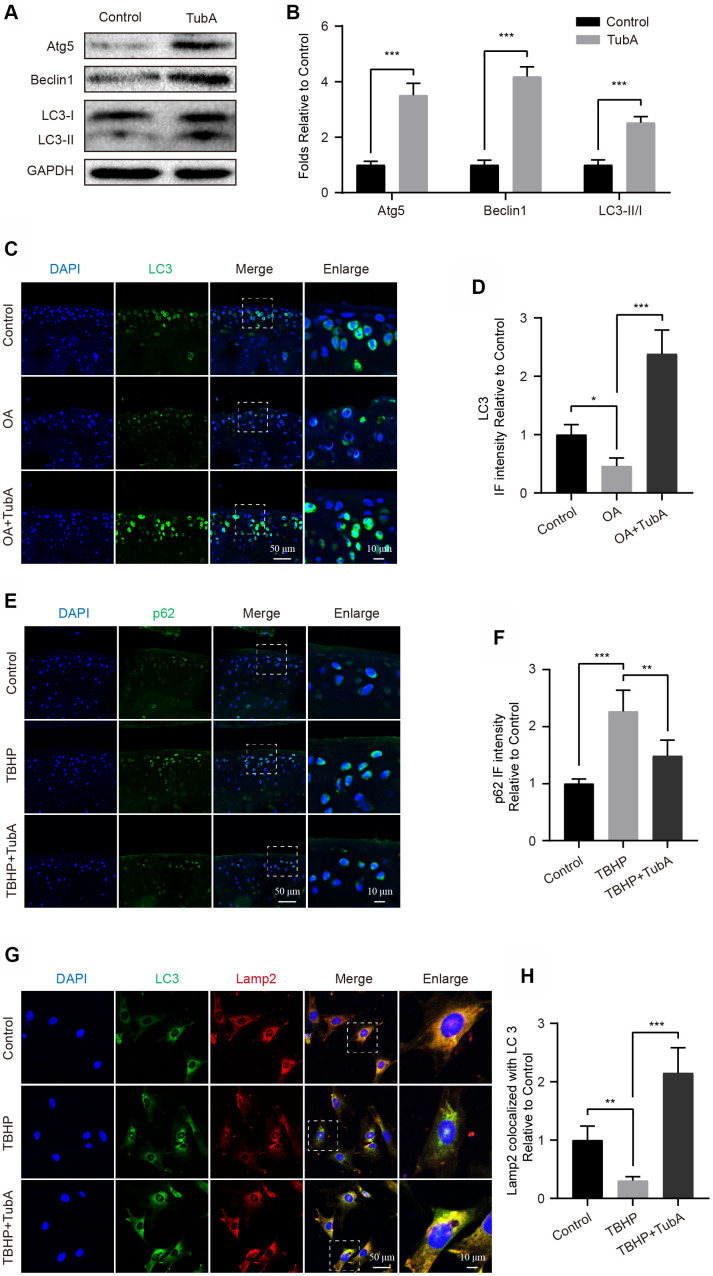
**Inhibition of HDAC6 by TubA activates autophagy.** (**A**, **B**) Western blotting and quantification of autophagic markers in each group. Chondrocytes were treated with TubA for 6 hours. (**C**, **D**) IF staining and quantification of LC3 in each mouse group, scale bar = 50 μm, scale bar (enlarged) = 10 μm. (**E**, **F**) IF staining and quantification of p62 in each mouse group, scale bar = 50 μm, scale bar (enlarged) = 10 μm. (**G**, **H**) Chondrocytes were treated with TBHP, TBHP + TubA for 6 hours, the co-localization of LC3 (green) and Lamp2 (red) were captured by IF staining and quantitatively analysed, scale bar = 50 μm, scale bar (enlarged) = 10 μm. N = 5, GAPDH was the loading control, significance: *P<0.05, **P<0.01, ***P<0.001.

Autophagy is a routine process for the removal of excess or damaged molecules in the development of ageing [19]. Regarding the beneficial effects of TubA on chondrocytes, we speculated that the underlying factor may be partly related to the modulation of autophagy. To evaluate the effect of TubA on chondrocyte autophagy, the level of autophagy relevant markers was measured by Western blotting. Like the results presented in Figure 5A, 5B, TubA treatment significantly increased the expression of Atg5, Beclin1 and LC3 II/I when compared with the control group. Furthermore, the increased LC3 (Figure 5C, 5D) and decreased p62 (Figure 5E, 5F) intensity were also observed in TubA-treated OA mice through staining with LC3 and p62, respectively. Considering that the fusion of autophagosome and lysosome is a critical process for the clearance of adverse molecules after the activation of autophagy, we analysed the colocalization of LC3 and Lamp2 in chondrocytes. The results from IF indicated that TBHP intervention causes a lower fusion dot of LC3 and Lamp2, whereas it is enhanced by TubA treatment (Figure 5G, 5H). Therefore, these results indicated that TubA activates the chondrocyte autophagy and may be involved in the beneficial effects.

### 3-MA reverses TubA-induced protection in chondrocytes

**Figure 6:**
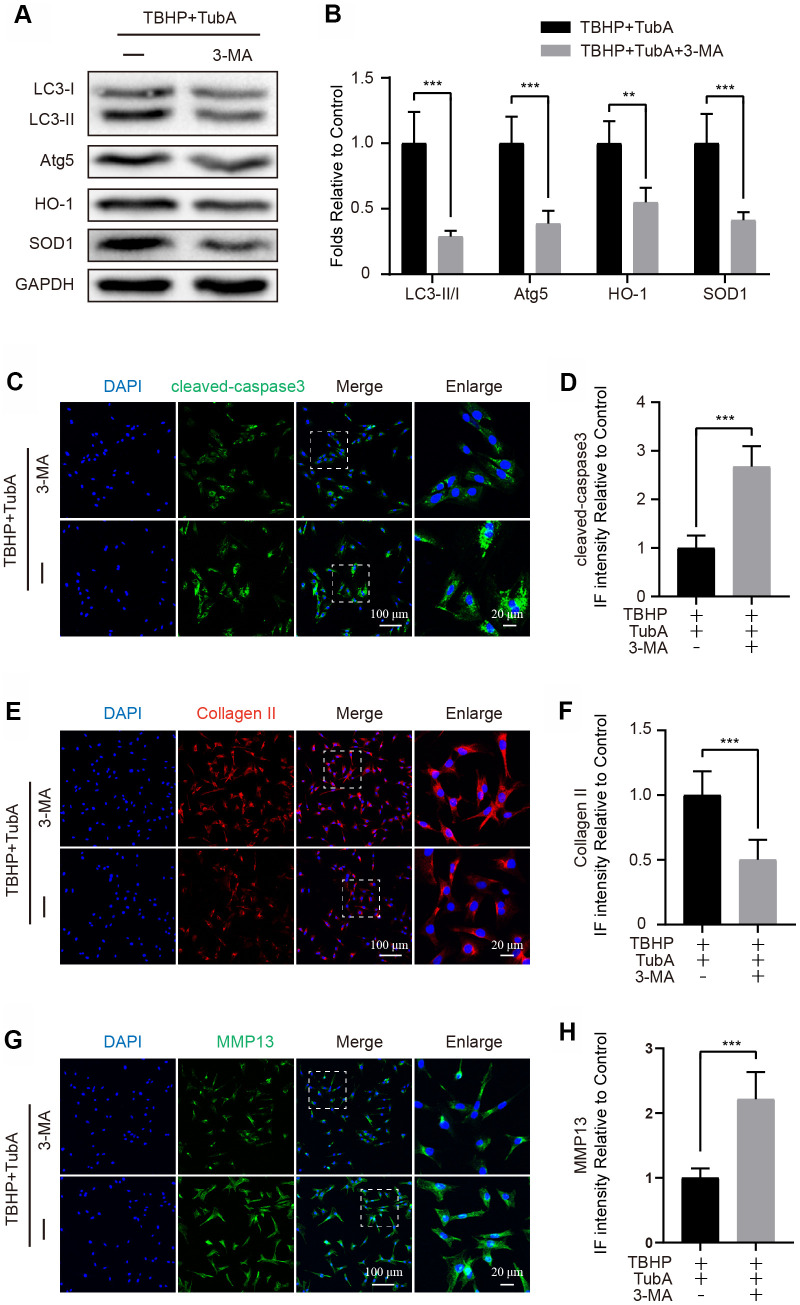
**3-MA treatment reverses the effects of TubA on oxidative stress, apoptosis and ECM degradation.** (**A**, **B**) Western blotting and quantification of LC3, Atg5, HO-1 and SOD1 in each group. Chondrocytes were treated with TBHP + TubA, or TBHP + TubA + 3-MA for 6 hours. (**C**, **D**) IF staining and quantification of cleaved caspase3 in each chondrocyte group as above, scale bar = 100 μm, scale bar (enlarged) = 20 μm. (**E**, **F**) IF staining and quantification of Collagen II in each chondrocyte group as above, scale bar = 100 μm, scale bar (enlarged) = 20 μm. (**G**, **H**) IF staining and quantification of MMP13 in each chondrocyte group as above, scale bar = 100 μm, scale bar (enlarged) = 20 μm. N = 5, GAPDH was the loading control, significance: *P<0.05, **P<0.01, ***P<0.001.

To determine whether TubA-activated autophagy is responsible for cell survival and ECM synthesis, chondrocyte was co-treated with TubA and 3-MA. First, to verify that 3-MA effectively suppresses autophagy in chondrocytes, the autophagy related markers were detected. The results from Western blotting demonstrated that3-MA intervention obviously decreased the expression of LC3II and Atg5 (Figure 6A, 6B). Thus, this result indicated that 3-MA successfully suppresses the autophagy activated by TubA in chondrocytes. Meanwhile, 3-MA co-administration also reduced the level of HO-1 and SOD1 (Figure 6A, 6B). However, as the results of IF showed, co-treatment with 3-MA and TubA also downregulated the intensity of cleaved caspase3 when compared with TubA treatment only (Figure 6C, 6D). Considering ECM, the results from IF showed that co-treatment of 3-MA remarkably decreased the Collagen II intensity (Figure 6E, 6F) but increased the MMP13 level (Figure 6G, 6H). Combining the above results, the advantageous effects of TubA are required for the activation of autophagy.

### TubA ameliorates OA in DMM mouse

**Figure 7:**
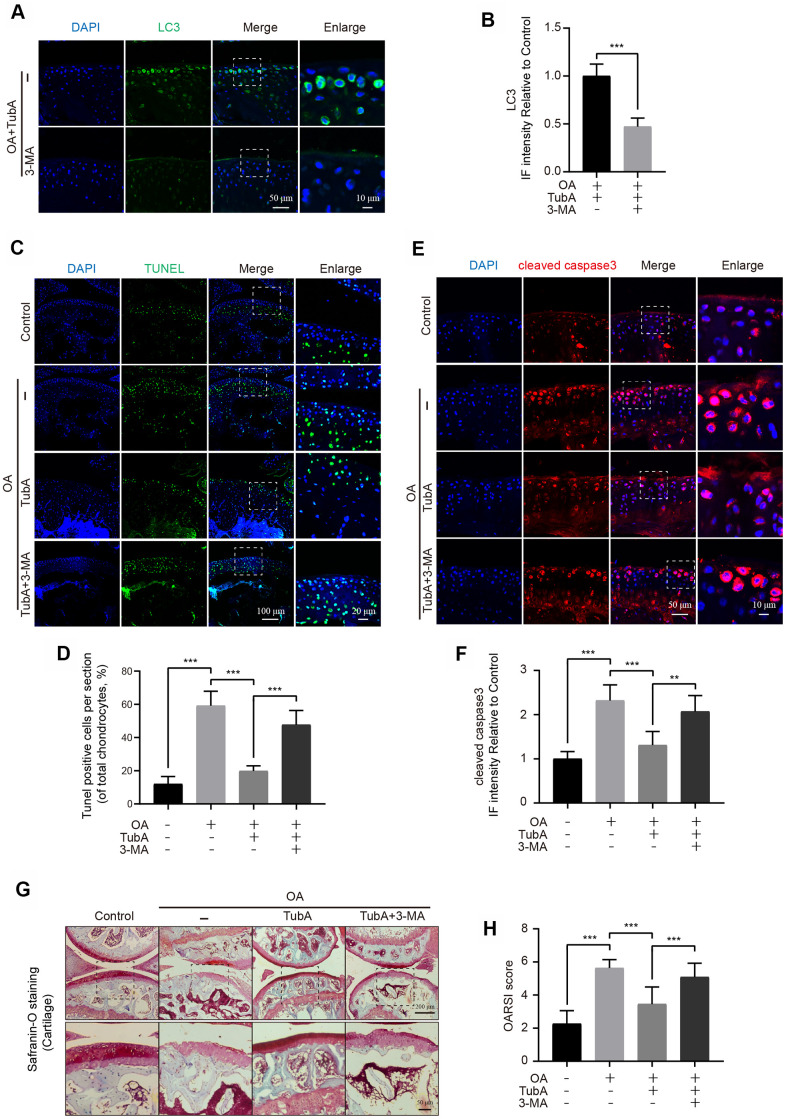
**TubA inhibits OA development in mice.** (**A**, **B**) IF staining and quantification of LC3 in each group of OA mice after treatment with TubA, 3-MA, scale bar = 50 μm, scale bar (enlarged) = 10 μm. (**C**, **D**) TUNEL staining quantification of positive cells in each group of mice, scale bar = 100 μm, scale bar (enlarged) = 20 μm. (**E**, **F**) IF staining and quantification of cleaved caspase3 in each group of mice as above, scale bar = 50 μm, scale bar (enlarged) = 10 μm. (**G**, **H**) Typical Safranin O staining of the cartilage and subchondral cortical bone in each group of mice, scale bar = 200 μm, scale bar (enlarged) = 50 μm. N = 5, GAPDH was the loading control, significance: *P<0.05, **P<0.01, ***P<0.001.

Finally, the potential therapeutic effect of HDAC6 inhibition by TubA on OA progression was assessed *in vivo*. The mice were treated with TubA, and experiments including TUNEL, Safranin O and IF staining were performed for evaluating histomorphology change in the mouse knee joints. Firstly, TubA treatment effectively decreased the expression of HDAC6 in the cartilage of OA mice (Supplementary Figure 2A, 2B). TUNEL staining revealed that the proportion of cellular apoptosis is lower in the TubA treatment group than in the OA group (Figure 7C, 7D). As the results showed in Figure 7E, 7F, the signal intensity of cleaved caspase3 was significantly reduced after administration of TubA compared with the OA group. Meanwhile, the results of Safranin O staining showed that the OA group exhibits the aberrance and hypocellularity of the superficial articular cartilage, while TubA reverses these pathological alterations and makes joints more integral and smooth (Figure 7G). Consistent with the Safranin O staining, the results of the OARSI were also decreased in the treatment group (Figure 7H). In addition, we also evaluated whether the effect of TubA is related to autophagy activation *in vivo*. IF showed that co-treatment with 3-MA effectively inhibits the level of LC3 in chondrocytes of the joint (Figure 7A, 7B). Like the results with chondrocytes *in vitro*, 3-MA intervention abolished the beneficial outcomes mediated by TubA on OA (Figure 7). Based on these findings, HDAC6 inhibition by TubA provides a protective effect on OA degeneration in DMM mice.

## DISCUSSION

**Figure 8:**
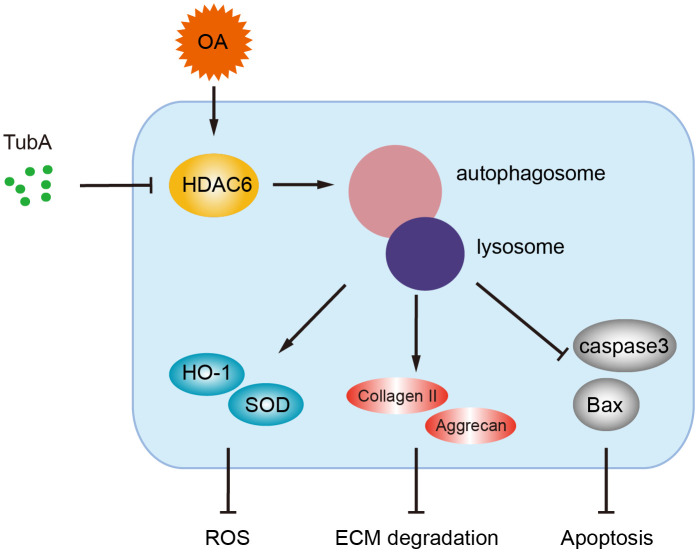
**A potential mechanism by which inhibition of HDAC6 by TubA decreases oxidative stress, apoptosis and ECM degradation.** Inhibition of HDAC6 by TubA preventing OA development is required for the activation of autophagy.

OA is a common joint degenerative disease that is related mainly to various pathological factor-induced joint cartilage surface damage. Nonsteroidal anti-inflammatory drugs (NSAIDs) are widely used in clinical drug treatment of OA. This kind of drug relieves OA symptoms only temporarily [30, 31]. Intriguingly, several pharmacological interventions or genetic regulations have been proven to be beneficial for maintaining chondrocyte homeostasis and preventing OA development [6–8]. In the current study, we have found that the level of HDAC6 is increased in OA mice, and pharmacological inhibition of HDAC6 by TubA alleviates cartilage damage. We also revealed that the therapeutic effect of HDAC6 inhibition by TubA is related to autophagy activation, which contributes to the attenuation of oxidative stress, reducing ECM degradation and inhibiting apoptosis (Figure 8). Here, we provide insight into the effects and underlying mechanisms of HDAC6 inhibition by TubA and present the potential value of TubA for OA in clinical treatment.

HDAC6, the only member of the HDAC family located in the cytoplasm, has the function of specifically catalysing the deacetylation of nonhistone substrates and participating in numbers of physiological or pathological mechanisms in various diseases [10–13]. HDAC6 was investigated mainly in cancer development and treatment at first and was believed to strongly relate to oncogenic cell transformation, cancer immunity regulation, increased cell mobility and mitosis. These studies demonstrated that inhibition of HDAC6 could be a potential and novel therapeutic strategy for cancer patients [10, 32, 33]. In addition, HDAC6 has been reported to be involved in acute cardiomyocyte injury [12], kidney disease [16], spinal cord injury [17], and stroke [34]. In addition, several studies also reveal that HDAC6 is associated with the development of age-related disease, including Alzheimer’s disease [35]. Though few studies have reported that HDAC6 is a potential determinant in the progression of OA, these existing studies could not allow a definite conclusion for elucidating the relationship between HDAC6 and OA. In line with previous studies, we also found that HDAC6 is up-regulated in the cartilage of OA mice and chondrocytes subject to TBHP.

As HDAC6 exerts such crucial regulating effects, several molecular inhibitors of HDAC6 have been introduced and applied for treatment. Based on molecular structure, the inhibitors were divided into four groups, including aliphatic acids, benzamides, cyclic peptides and hydroxamates [26]. In the current study, we selected TubA, a potent and selective inhibitor, for repressing HDAC6 expression. As proved previously, TubA has been shown to contribute to the recovery from spinal cord injury, as well as treatment of rheumatism and autoimmune disorders [17, 36, 37]. Consistent with previous studies, our findings suggested that HDAC6 could be effectively inhibited by TubA in chondrocytes, and it possesses the capability of preventing OA development. However, previous studies have reported that HDAC6 inhibitor may cause a series of toxic and adverse effects owing to use of an inadequate dose. Here, according to the use rules of previous studies, the current dose of TubA results in no obvious cell death in cell viability experiments, both in time- and dose-dependent manners.

In consideration of ROS, apoptosis and ECM degradation play an important role in the progression of OA. The effect of HDAC6 inhibition on the above molecular events was evaluated. As an age-related degenerative disease, the abnormal accumulation of ROS production was a common pathophysiology in OA development, and clearing the excessive ROS was helpful for reducing damage to DNA and members of organelles [4, 19]. HDAC6 inhibition has been reported to contribute against oxidative stress by increasing the level of antioxidant enzymes [38]. Currently, the positive effect of HDAC6 inhibition on regulating oxidative stress has also been proven. Our findings suggested that HDAC6 inhibition by TubA effectively enhances the expression of HO-1 and SOD1, which are two well-known antioxidant enzymes in the cytoplasm. Meanwhile, the aberrant accumulation of ROS could damage the redox reaction homeostasis and result in oxidative apoptosis. In line with this notion, the evidence of the current study also revealed that HDAC6 inhibition represses the expression of apoptotic proteins and maintains chondrocyte survival. In addition, previous studies have reported that ROS is a negative factor in the regulation of ECM metabolism. Oxidative stress-induced ECM degradation is one of the reasons for accelerating joint degeneration [39]. Like previous reports, HDAC6 inhibition improved the ECM-related enzyme expression and reversed the adverse ECM degradation.

Numerous studies have highlighted an important role of autophagy in chondrocyte homeostasis and cartilage morphogenesis [4, 19]. At a low rate of cell turnover in an avascular organ, previous studies showed that chondrocytes adapt to the low energy expenditure situation dependent on autophagy activation. However, the existent studies indicated that the autolysosome formation failed due to the declining autophagy function and impaired lysosome activity in OA [4, 19]. As previously described, HDAC6 is an essential regulator for autophagy. Song et al. [12] indicated that HDAC6 inhibition by TubA protected against the Doxorubicin-induced acute cardiomyopathy required for α-tubulin acetylation-mediated autophagy activation. Another study reported by Brijmohan et al. [16] suggested HDAC6 inhibition enhances autophagy by promoting a transcription factor termed transcription factor EB (TFEB), which is responsible for TubA-mediated protection in chronic kidney disease. We presumed that HDAC6 inhibition-mediated protection in OA is dependent on autophagy activation. In our present work, HDAC inhibition by TubA increases the expression of autophagy related proteins and promotes the fusion of autophagosomes and lysosomes, which is responsible for clearing ROS, promoting cell survival and inhibiting ECM degradation evidenced by the administration of 3-MA.

Certainly, some limitations of the current study should be noted and still requires further investigation. For instance, the underlying mechanism of HDAC6-regulated autophagy has not been well investigated. Previous studies revealed that TFEB regulation and α-tubulin were involved in the HDAC6-mediated autophagy [12, 16]. Meanwhile, HDAC has been reported to directly affect the post-translational acetylation of various autophagy related proteins, including p62 acetylation [23]. Therefore, we speculated that HDAC6-mediated autophagy is a result of multiple mechanisms. In addition, a single dose of TubA for OA mice performed directly after DMM with optimal dose and duration time of treatment will obtain a better understanding and still requires further study. Furthermore, though the selective inhibition of HDAC6 was applied, we admit that TubA may be affecting other forms of HDAC6. The protective effect entirely attributed to the function of HDAC6 on autophagy may be one-sided. Therefore, employing virus vector or gene knockdown mice instead of drug administration would be more persuasive.

In conclusion, the current study suggested that the expression of HDAC6 is elevated in OA mice. Inhibition of HDAC6 by TubA activates autophagy and enhances autolysosome formation, sequentially attenuating oxidative stress, inhibiting apoptosis and reducing ECM degradation in chondrocytes and preventing OA development in mice. Collectively, the inhibition of HDAC6 by TubA exhibits a novel potential therapeutic strategy for the management of OA.

## MATERIALS AND METHODS

### Animals and ethics statement

Adult male C57BL/6 mice (7-8 weeks old, 20-25 g) were obtained from Jiaxing University and housed in the Experimental Animal Cage System under standard condition with the temperature at 23 ± 2° C, humidity at50 ± 5% and 12 h light/dark cycle. All experimental performance, reagent treatments, and postoperative animal care procedures were strictly conducted in accordance with the Animal Care and Use Committee of Jiaxing University. No clinical trial was involved in the current study.

### Reagents and antibodies

Reagents including TubA (HY-13271A, purity≥98%) were purchased from MedChemExpress (NJ, USA), TBHP solution (416665) and 3-methyladenine (3-MA, M9281) were obtained from Sigma-Aldrich Chemical Company (WI, USA), 4,6-Diami-dino-2-phenylindole (DAPI, P0131) was purchased from Beyotime (Shanghai, China). Primary antibodies were included as the following: HDAC6 (12834–1), GAPDH (10494-1), Bcl-2 (26593-1), HO-1 (10701-1), SOD1 (10269-1), Beclin 1 (11306-1), Aggrecan (13880-1), MMP13 (18165-1) and α-tubulin (66031-1) from Proteintech (IL, USA); Bax (ab182733), Collagen II (ab185430), ADAMTS5 (ab41037), Lamp2 (ab13524) and p62 (ab211324) from Abcam (MA, USA); ATG5 (12994) and acetyl-α-tubulin (5335) from CST (MA, USA); cleaved caspase 3 from (AF7022) from Affinity Biosciences (OH, USA); LC3B (NB600-1384) from NOVUS Biologicals (CO, USA); secondary antibodies against mouse, rat and rabbit were purchased from Proteintech; Alexa Fluor FITC (488) or Cy5 (647) donkey anti-rabbit/mouse/rat secondary antibodies were purchased from Abcam.

### Establishment of an OA model and group setting

The osteoarthritis model was performed by surgical DMM as previously introduced [6]. In brief, a total of forty-five mice were included and intraperitoneally injected with 2% (w/v) pentobarbital (40 mg/kg) for anaesthesia. Using microsurgical scissors, the joint capsule, medial meniscotibial ligament of the right mouse knee was transected sequentially, and the knee joint performing with arthrotomy without the transaction of the medial meniscotibial ligament was regarded as the control group. After surgical intervention, the mice were randomly divided into four groups: control group, DMM group, DMM + TubA group, and DMM + TubA + 3-MA group. The related drug administration was as follows: TubA (50mg/kg/day) was injected intraperitoneally for inhibiting HDAC6 expression, and 3-MA (10 mg/kg) was injected intraperitoneally for inhibiting autophagy. The control group and the DMM group received equivalent doses of normal saline. All drugs were used consecutively until mice were sacrificed.

### Tissue preparation

Mice were sacrificed by an overdose of 8% pentobarbital (40 mg/kg) at 8 weeks after surgery in each group. The knee joints were harvested and fixed in 4% (v/v) paraformaldehyde (PFA) for 24h. Then, the joints were decalcified in 10% (v/v) ethylenediaminetetraacetic (EDTA) for 4 weeks, and the EDTA was replaced with fresh every 2 days. For subsequent staining analysis, the specimens were dehydrated, embedded in paraffin and cut into 0.5-μm slides by sagittal section.

### Primary chondrocyte culture

Primary chondrocytes were obtained from postnatal Day 0 (P0) mouse pups by dissecting the knee cartilages as previously described [6]. In brief, cartilages were cut into pieces and digested with 2mg/mL of collagenase II in DMEM/F12 medium at 37° C for 4h. By washing and separating the suspension, chondrocytes were collected and seeded on plate in DMEM/F12 medium with 10% foetal bovine serum (FBS), 100U/mL penicillin, and 100 μg/mL streptomycin at 37° C in a 5% CO_2_-containing atmosphere. The media were replaced with fresh after the first 24 hours of incubation. When up to 70-80% confluency is achieved, chondrocytes were harvested by 0.25% Trypsin-EDTA (12605028, Gibco, California, USA) and re-seeded on a plate at the appropriate density. The second passage cells were seeded on 12 well plates, employed for all our experiments, and replaced with fresh media every 2 days thereafter until the experiments were finished.

### Cell viability assay

Cell viability was evaluated by a CCK-8 assay (CK04, Dojindo Laboratories, Kyushu, Japan) according to the manufacturer’s protocol. Briefly, the chondrocytes were digested by 0.25% Trypsin-EDTA, seeded on a 96-well plate (1x10^4^ cells per well), and incubated in 5% CO_2_ at 37° C with complete medium for 24 hours. Then, the chondrocytes were treated with drugs as designed. After drug treatment, 10 μL of CCK-8 solution was added to each well and cultured for 2 hours. Absorbance was measured at 450 nm using a microplate reader.

### Measurement of ROS generation and mitochondrial membrane potential

To detect intracellular ROS generation, a 2′,7′-dichlorofluorescin diacetate fluorescent probe (DCFH-DA, S0033S, Beyotime) was applied according to manufacturer’s instructions. In brief, chondrocytes were seeded on 12-well plate (1×10^6^/well), pretreated with or without TubA, and subjected to TBHP for 6 hours. After drug administration, the medium was replaced with DMEM/F12 containing 15 μM DCFH-DA for 30 minutes at the incubator with 37° C and 5% CO_2_. Sequentially, chondrocytes were washed with PBS three times for removing unwanted DCFH-DA and immediately put on the worktable of the Nikon ECLIPSE Ti microscope (Nikon, Tokyo, Japan) for image capture. The fluorescence intensity was analysed by ImageJ software (version1.52p). The mitochondrial membrane potential assay was evaluated by the Mitotracker Green fluorescent probe (C1048, Beyotime) according to the manufacturer’s instructions. Chondrocytes were incubated as above, treated with 100 nM Mitotracker Green for 30minutes at 37° C and then labelled with DAPI for 5 minutes. The images were also visualized by the Nikon ECLIPSE Ti microscope, and the intensity was measured by ImageJ software.

### Western blotting

The chondrocyte proteins were separated by RIPA lysis buffer and quantified by Bradford. A total of 40 μg of protein was separated on SDS-PAGE and transferred onto a PVDF membrane. Then, the PVDF membranes were blocked with 5% (w/v) skimmed milk for 90 minutes at room temperature and sequentially incubated with primary antibodies overnight at 4° C. The titre of primary antibodies was listed: HDAC6 (1:500), cleaved caspase3 (1:1000), Bax (1:500), Bcl2 (1:500), Collagen II (1:1000), ADAMST5 (1:1000), Aggrecan (1:1000), Atg5 (1:1000), Beclin1 (1:1000), LC3 (1:1000), HO-1 (1:1000), SOD1 (1:1000), GAPDH (1:1000), α-tubulin (1:1000), acetyl-α-tubulin (1:1000). On the second day, the PVDF membranes were washed and incubated with secondary antibodies for 120 minutes at room temperature. Last, the grey value of PVDF membranes were visualized by ChemiDoc XRS+ Imaging System (Bio-Rad, CA, USA) and measured by Quantity-One software (Version 4.6.9). Measurements were obtained from at least five samples.

### Immunofluorescence staining

The deparaffinized and rehydrated joint sections were treated with EDTA for antigen restoration, and the cultured chondrocytes were fixed with 4% PFA. Then, both were blocked with 5% (w/v) bovine serum albumin for 30 minutes at 37° C. After washing by PBS, sections were incubated with primary antibodies overnight at 4° C, the titre of primary antibodies was listed: HDAC6 (1:300), cleaved caspase3 (1:1000), MMP13 (1:300), Collagen II (1:500), LC3 (1:1000), Lamp2 (1:1000), p62 (1:1000). Next, sections were washed and stained with Alexa Fluor FITC or Cy5 conjugated secondary antibodies for 90 minutes at 37° C, and then stained with DAPI for 7 minutes. The results were captured by a Nikon ECLIPSE Ti microscope and analysed by ImageJ software. Measurements were obtained from five random sections, with three random visual fields each.

### TUNEL staining and histopathologic analysis

A terminal deoxynucleotidyl transferase dUTP nick-end labelling (TUNEL) assay was applied for apoptotic analysis. According to the manufacturer’s instructions, sections were deparaffinized, rehydrated and incubated with 0.1% Triton X-100 for 30 minutes. The sections of chondrocyte were fixed with PFA and washed with PBS. Next, sections were incubated with 50 μL *In Situ* Cell Death Detection Kit (40307ES60, Yeasen Biochemical, Shanghai, China) and stained with DAPI for 7 minutes. The images were also captured by a Nikon ECLIPSE Ti microscope. The histopathologic analysis was detected by the safranin O-fast green (S-O) staining. Using an Osteoarthritis Research Society International (OARSI) scoring system [40], the cellular structure and morphology of cartilage and subchondral bone from S-O staining were examined by experienced histology researchers in a blinded manner.

### Statistical analysis

Statistical analyses were performed using GraphPad Prism (Version 8.0.2). All the data are presented as the mean ± SEM from at least three independent experiments. Statistical analysis was performed using Student's t-test or the Mann-Whitney rank sum test for two-group comparison, one way analysis of variance (ANOVA) followed by a Tukey's test or Kruskal-Wallis ANOVA based on ranks followed by Dunn’s post hoc test for pairwise comparisons of multiple groups. A p value < 0.05 indicated significance.

### Data availability

The datasets used and analysed during the current study are available from the corresponding authors on reasonable request.

## Supplementary Material

Supplementary Figures
