# Supplementary material for: Inhibition of HDAC6 by Tubastatin A reduces chondrocyte oxidative stress in chondrocytes and ameliorates mouse osteoarthritis by activating autophagy
Source: Aging (Albany NY). 2021 Mar 19;13(7):9820–37. doi: 10.18632/aging.202736 (PMC8064156; doi:10.18632/aging.202736)
Supplement: Supplementary Figures [file aging-13-202736-s001.pdf]

SUPPLEMENTARY FIGURES

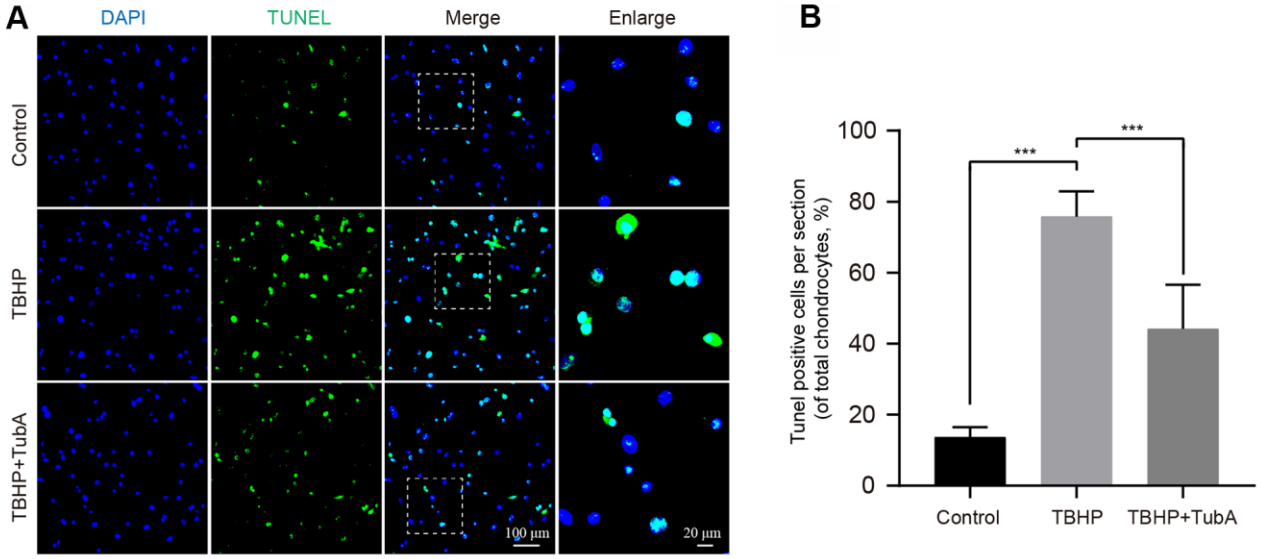

Supplementary Figure 1. Inhibition of HDAC6 by TubA prevents TBHP-induced apoptosis in chondrocytes. (**A**, **B**) TUNEL staining quantification of positive cells in each group of chondrocytes, scale bar = 100 µm, scale bar (enlarged) = 20 µm.

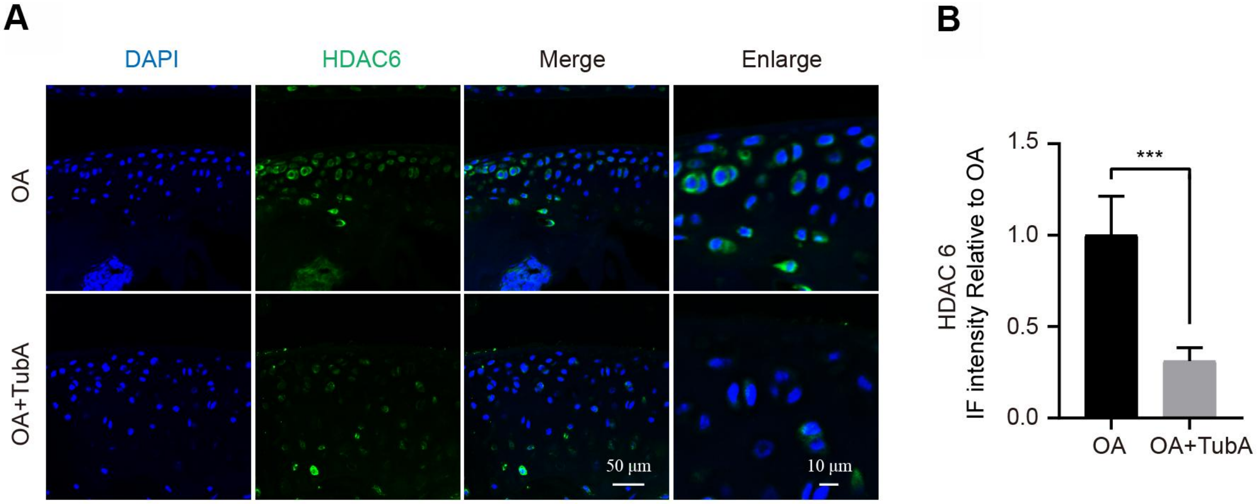

Supplementary Figure 2. TubA treatment decreases HDAC6 expression in OA mice. (**A**, **B**) IF staining and quantification of the changes in HDAC6 in each group of mice, scale bar = 50 µm, scale bar (enlarged) = 10 µm.
